# S-palmitoylation and depalmitoylation at the interface of animal virus-host interactions

**DOI:** 10.3389/fcimb.2026.1773311

**Published:** 2026-03-04

**Authors:** Rui-Qin Xu, Xi-Meng Chen, Chen-Rong Wang, Shi-Jie Ma, Ping-Li Wang, Hong-Ying Chen

**Affiliations:** 1 College of Veterinary Medicine, Henan Agricultural University, Zhengzhou, Henan, China; 2 School of Modern Agriculture and Biotechnology, AnKang University, Ankang, Shanxi, China; 3 Henan Province Key Laboratory for Animal Food Pathogens Surveillance, Zhengzhou, Henan, China; 4 Ministry of Education Key Laboratory for Animal Pathogens and Biosafety, Zhengzhou, Henan, China

**Keywords:** depalmitoylation, immune evasion, signal pathway, S-palmitoylation, viral lifecycle

## Abstract

Reversible protein S-palmitoylation, mediated by protein acyl transferases (PATs) and depalmitoylases, is essential for regulating numerous biological processes, including subcellular localization, protein stability, enzymatic activity, and protein-protein interactions. While the study of S-palmitoylation in virology is extensive, less attention has been paid to its reverse process, depalmitoylation, at the virus-host interface. This review summarizes the dynamic regulatory mechanisms of both S-palmitoylation and depalmitoylation. We systematically review the functional consequences of these host enzyme-mediated modifications based on the roles of viral proteins in the viral life cycle. Next, we focus on how viruses exploit these modifications for immune evasion and the corresponding host antiviral strategies. Finally, we analyze the distinct role of depalmitoylation in regulating viral replication and host defense. Overall, this review aims to provide new insights into the regulatory mechanisms of reversible S-palmitoylation at the virus-host interface.

## 1 Introduction

Viruses in the environment pose significant risks to both humans and animals, as they can cause infections that may lead to the host’s death. This review will focus on representative animal viruses, including those of significant zoonotic potential that pose threats to both human and animal health. As obligate intracellular parasites, viruses must hijack the host’s cellular machinery for their replication, which is vital for their survival and growth. Moreover, viral hijacking often converges on key host molecular modification systems that regulate core physiological functions, making them focal points in the virus-host interplay. A typical example is the dynamic cycle of S-palmitoylation and depalmitoylation. By controlling protein membrane targeting, protein-protein interactions, and stability, this reversible post-translational modification (PTM) is integral to cellular signaling and immunity, positioning it as a crucial interface for viral invasion and host defense. Viruses actively co-opt the host S-palmitoylation apparatus to promote steps in their life cycle, from attachment to progeny release. Conversely, the host leverages the S-palmitoylation/depalmitoylation mechanism to trigger antiviral innate immune responses and curb viral spread.

Protein S-palmitoylation is a reversible lipid modification widespread in eukaryotic cells, involving the attachment of a 16-carbon palmitoyl group to the cysteine residues of proteins via a thioester bond, as well as the reverse process of depalmitoylation. The initial discovery of this modification dates back to 1979, when Schmidt and colleagues reported the palmitoylation of the vesicular stomatitis virus (VSV) glycoprotein, revealing a novel form of post-translational modification (Schmidt and Schlesinger, 1979). The palmitoyl moiety used for this modification is derived from cellular metabolism: palmitic acid is first synthesized *de novo* by fatty-acid synthase (FAS), then activated to palmitoyl coenzyme A (Pal-CoA) by acyl-CoA synthetase, and finally serves as the immediate donor substrate for palmitoyltransferases.

Specifically, S-palmitoylation is catalyzed by a family of zinc finger DHHC domain-containing (ZDHHC) palmitoyltransferases, which includes ZDHHC1–9 and ZDHHC11-24 (Elliot Murphy and Banerjee, 2022). The reverse reaction, depalmitoylation, is catalyzed by depalmitoylases, such as the acyl protein thioesterase (APT) family (APT1/2), the palmitoyl protein thioesterase (PPT) family (PPT1/2), and the alpha/beta-hydrolase domain-containing (ABHD) family (ABHD17A/B/C, ABHD10, and ABHD16A) (Shi et al., 2022; Cai et al., 2023; Fan et al., 2023; Wang et al., 2024). The complete palmitoylation/depalmitoylation cycle occurs within seconds to hours (Won and Martin, 2018). Under physiological conditions, palmitoyltransferases work together with depalmitoylases to regulate the level of S-palmitoylation on target proteins. This process contributes to a wide range of biological functions, including protein subcellular localization, stability, and trafficking, thereby participating in processes such as cell growth, differentiation, stress response, and immune regulation. Numerous studies have increasingly demonstrated that the processes of S-palmitoylation and depalmitoylation are significantly linked to the development of several human diseases, including cancer, neurological conditions, cardiovascular issues, infectious diseases, and immune-related disorders (Jin et al., 2021; Wang et al., 2024). Consequently, palmitoyltransferases, depalmitoylases, and their specific substrate proteins, represent promising avenues for disease treatment.

In the context of animal viral infection, viruses can hijack S-palmitoylation to modify their own proteins, thereby facilitating multiple stages of the infection cycle, such as entry, replication, assembly, and release. Conversely, the host also utilizes this modification to regulate antiviral innate immune signaling pathways and establish an antiviral defense. While recent reviews have summarized the functions of ZDHHCs in viral infection (Gadalla and Veit, 2020; Yi and Zheng, 2021; Li et al., 2022), these studies primarily focus on the enzymatic machinery of palmitoylation and its role in host antiviral immunity. A comprehensive review specifically focusing on the dynamic interplay between palmitoylation and depalmitoylation across various viruses is still lacking. Notably, depalmitoylases, including the APT, PPT, and ABHD families, have received limited attention in the context of viral infection, despite their critical role in maintaining the dynamic equilibrium of protein S-palmitoylation. Here, we provide a balanced perspective on how both arms of this reversible modification regulate the viral life cycle (entry, replication, assembly, and release), host antiviral immune responses (cGAS-STING, TRAF6-TAK1, IFITM, and MAVS pathways), and viral immune evasion strategies. By integrating recent findings on both palmitoyltransferases and depalmitoylases, this review aims to advance our understanding of the regulatory dynamics governing protein S-palmitoylation in virus-host interactions.

## 2 Enzymes and mechanisms of reversible S-palmitoylation

The S-palmitoylation and depalmitoylation cycle constitutes a rapid, dynamic, and reversible regulatory switch that is responsible for regulating protein function in response to cellular stimulation, especially viral infection. This cycle is catalyzed by dedicated enzymatic machineries, which include palmitoyltransferases and depalmitoylases, whose coordinated actions precisely regulate the membrane association, trafficking, and stability of a vast array of substrate proteins. Understanding the biochemical mechanism of this cycle is a key to understanding its multifaceted roles in viral pathogenesis and host immunity.

### 2.1 ZDHHCs

In mammalian cells, protein S-palmitoylation is primarily catalyzed by the DHHC family of palmitoyltransferases. These enzymes are multi-pass transmembrane proteins, typically containing 4 to 6 transmembrane domains (TMDs), with their N- and C-termini oriented toward the cytosol. A key characteristic of this family of enzymes is the highly conserved catalytic core between the second and third transmembrane helix. This core contains the catalytic motif aspartate-histidine-histidine-cysteine (DHHC), where cysteine acts as the active nucleophile engaging in auto palmitoylation (Mitchell et al., 2006; Gottlieb and Linder, 2017). Critically, within this DHHC motif, two zinc ions (Zn^2+^) are coordinated to form a folded zinc finger domain. As this zinc-binding property is fundamental to the domain’s stability and function, the protein family is also named ZDHHC proteins (González Montoro et al., 2013). Genomic databases for humans and mice indicate that this family comprises 23 members, including ZDHHC1-ZDHHC9 and ZDHHC11-ZDHHC24, which encode 23 distinct proteins (Elliot Murphy and Banerjee, 2022). Additionally, some family members, such as ZDHHC13 and ZDHHC17, possess an N-terminal ankyrin repeat domain that specifically mediates interactions with substrate proteins (Lemonidis et al., 2014). Furthermore, most ZDHHC proteins are localized to the endoplasmic reticulum and Golgi apparatus, while a subset, including ZDHHC5, ZDHHC20, and ZDHHC21, predominantly reside at the plasma membrane (Rocks et al., 2010). This distinct subcellular localization determines the subset of substrates accessible to each enzyme, thereby spatially regulating protein S-palmitoylation across different cellular compartments.

### 2.2 Depalmitoylases

Currently, ten depalmitoylases have been identified, which are classified into three main families: 1) the acyl protein thioesterase (APT) family, including APT1/LYPLA1 and APT2/LYPLA2, which are primarily cytosolic; 2) the palmitoyl protein thioesterase (PPT) family, comprising PPT1 and PPT2, which are predominantly localized to lysosomes; and 3) the alpha/beta-hydrolase domain-containing (ABHD) family, including ABHD10 (mitochondria), ABHD16A (membranous), and ABHD17A/B/C (plasma membrane) (Shi et al., 2022; Cai et al., 2023; Fan et al., 2023; Wang et al., 2024). All these enzymes belong to the serine hydrolase superfamily. Despite variations in their overall structure, all depalmitoylases contain a conserved α/β-hydrolase fold and a catalytic triad, which enables them to efficiently catalyze depalmitoylation reactions. Notably, in contrast to S-palmitoylation, which relies on consensus sequences like the DHHC motif, no strict sequence specificity has been identified for depalmitoylase substrate recognition so far (Rocks et al., 2010).

### 2.3 Catalytic mechanism of S-palmitoylation and depalmitoylation

**Figure 1:**
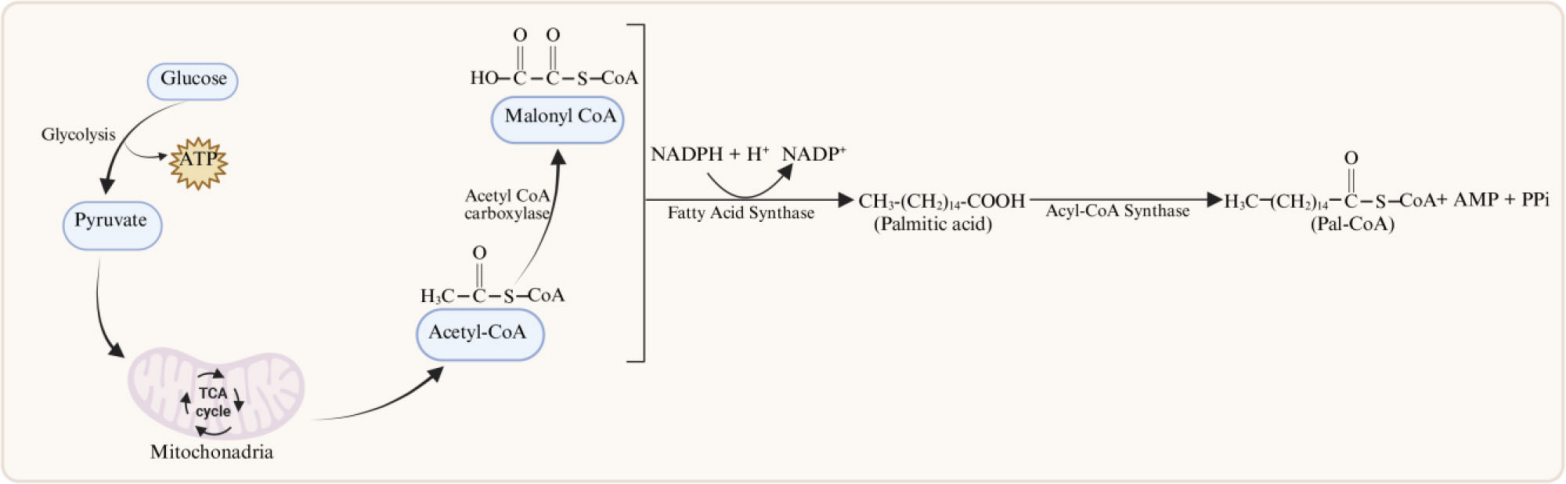
Biosynthesis of Pal-CoA. Pal-CoA, the lipid donor for protein S-palmitoylation, is primarily generated through the *de novo* synthesis pathway mediated by host fatty acid synthase (FAS). This process is initiated by the cellular uptake of glucose, which is then metabolized through glycolysis to yield pyruvate. Within the mitochondria, pyruvate is converted into acetyl-CoA in a reaction catalyzed by pyruvate dehydrogenase. The resulting acetyl-CoA is transported to the cytosol, where it serves as a substrate for acetyl-CoA carboxylase—the rate-limiting enzyme that catalyzes the formation of malonyl-CoA. Using acetyl-CoA as the primer and malonyl-CoA as the two-carbon extender, FAS catalyzes a repetitive cycle of condensation, reduction, dehydration, and a second reduction. Each turn of this cycle elongates the fatty acyl chain by two carbon atoms, ultimately leading to the synthesis of the 16-carbon fatty acid, palmitic acid. Finally, palmitic acid is converted to pal-CoA by acyl-CoA synthetase. Created in BioRender. ruiqin, X. (2026) https://BioRender.com/j3wwu0q is licensed under CC BY 4.0.

S-palmitoylation and depalmitoylation constitute a dynamic, reversible modification that critically regulates cellular adaptation to changing physiological conditions and external stimuli. The immediate donor of the palmitoyl group is Pal-CoA. Pal-CoA is generated through the acyl-CoA synthetases–mediated activation of palmitic acid, which is primarily produced through *de novo* lipogenesis (**Figure 1**). This pathway begins with glucose uptake and glycolysis; the generated pyruvate is converted to acetyl-CoA by pyruvate dehydrogenase in mitochondria. After export to the cytosol, acetyl-CoA is carboxylated to malonyl-CoA by acetyl-CoA carboxylase. Then palmitic acid is synthesized by FAS through a series of reactions that sequentially condense acetyl-CoA with malonyl-CoA.

**Figure 2:**
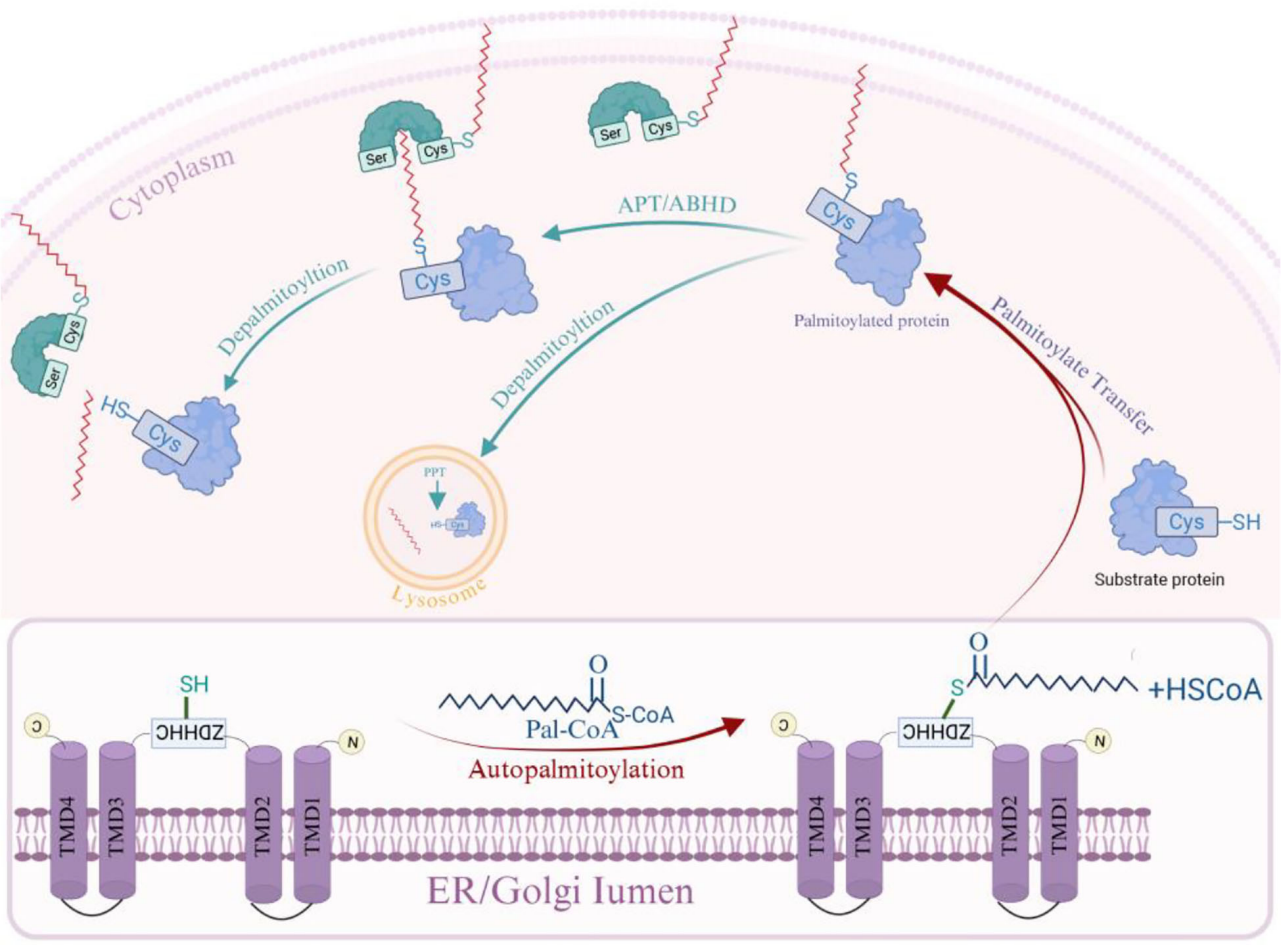
Protein S-palmitoylation/depalmitoylation cycle. Palmitate from Pal-CoA is first linked via a thioester bond to the ZDHHC motif of a palmitoyltransferase, resulting in auto-palmitoylation of the enzyme; the palmitate moiety is then transferred to a specific cysteine residue of the substrate protein in a reaction mediated by the palmitoyltransferase. This modification is reversible; the thioester bond can be hydrolyzed by specific depalmitoylases (such as certain APT, ABHD, or PPT). Created in BioRender. ruiqin, X. (2026) https://BioRender.com/dz8h45j is licensed under CC BY 4.0.

Palmitoyltransferases exploit the resulting Pal-CoA to attach palmitate to target proteins through a two-step mechanism (**Figure 2**): first, the enzyme undergoes auto-palmitoylation, whereby a cysteine residue in the palmitoyltransferase utilizes Pal-CoA derived from cellular metabolism to form an acyl-enzyme intermediate via a thioester bond, releasing free CoA-SH. In the absence of a substrate protein, the resulting intermediate is inherently unstable and undergoes slow auto-hydrolysis. Second, in the presence of a substrate protein, the palmitoyl group is transferred from the intermediate to a cysteine residue on the substrate, thereby achieving S-palmitoylation of the target protein (Stix et al., 2020).

Depalmitoylation is catalyzed by depalmitoylases via hydrolysis (**Figure 2**). All these depalmitoylases share a core α/β-hydrolase fold and a catalytic triad. The catalytic triad activates a water molecule, which then nucleophilically attacks the thioester bond linking the palmitic acid to the cysteine residue of the substrate protein. This hydrolysis reaction cleaves the bond, releasing free palmitate and the depalmitoylated protein. Understanding the enzymes and mechanisms of reversible S-palmitoylation is key to explaining how viruses hijack this system and how hosts exploit it for immune defense.

## 3 S-palmitoylation of viral protein

S-palmitoylation is a dynamic and reversible lipid modification that is widely exploited by diverse viruses to orchestrate multiple key stages of their life cycle. Despite their genetic diversity, viruses lack genes encoding palmitoyltransferases and therefore depend on the host enzymes for this modification. In this section, we systematically review how viruses strategically leverage S-palmitoylation to facilitate each successive step of their life cycle and to immune evasion. The functional consequences of viral protein S-palmitoylation are multifaceted, primarily involving the modulation of membrane affinity, ensuring correct subcellular trafficking, stabilizing protein complexes, and promoting virus-host membrane interactions (as summarized in **Table 1**). We will follow the viral life cycle, beginning with the role of S-palmitoylation in entry and fusion proteins of enveloped and non-enveloped viruses, proceeding to its critical functions in anchoring replication complexes, and culminating in its contributions to viral assembly, release, and finally, the subversion of host immune responses.

**Table 1:** S-palmitoylation of viral proteins and their functional roles in the virus life cycle.

Family	Genus	Virus	Protein name	ZDHHCs	Major functions & mechanisms of palmitoylation	Refs.
Orthomyxoviridae	Influenza A virus	IAV	HA	ZDHHC2, 8, 15, 20	Promotes lipid raft localization, membrane fusion, and virus assembly.	(Siche et al., 2015; Gadalla et al., 2020)
	Influenza B virus	IBV	HA	ZDHHC1, 2, 4, 6	Essential for virus replication; affects early replication stages.	(Meng and Veit, 2023)
	Influenza C virus	ICV	HEF	–	S−palmitoylation of HEF occurs at a negligible level and fails to mediate its raft localization.	(Veit et al., 2013; Gadalla et al., 2020)
Coronaviridae	Alphacoronavirus	TGEV	S	–	Essential for incorporation into virus-like particles (VLPs); dispensable for S-M interaction.	(Gelhaus et al., 2014)
		PEDV	S	–	Enhances protein stability by antagonizing chaperone-mediated autophagy degradation.	(Qian et al., 2025)
	Betacoronavirus	MHV	S	–	Essential for virion assembly and infectivity; promotes S-M interaction.	(Thorp et al., 2006)
		SARS-CoV-2	S	ZDHHC2, 3, 4, 5, 6, 8, 9, 11, 14, 16, 19, 20, 21, 24 (ZDHHC20 key)	Essential for membrane fusion, syncytia formation, and virus entry; regulates trimer stability.	(Mesquita et al., 2021; Puthenveetil et al., 2021; Zeng et al., 2021; Li et al., 2022; Tien et al., 2022)
		SARS-CoV	S	–	Essential for membrane fusion; targets membrane domains; has no effect on S protein stability, localization, or trafficking.	(Akerström et al., 2009; McBride and Machamer, 2010)
Retroviridae	Lentivirus	HIV-1	gp41 (Env)	–	Promotes lipid raft association, virion incorporation, and infectivity.	(Yang et al., 1995; Rousso et al., 2000; Bhattacharya et al., 2004; Chan et al., 2005; Alfadhli et al., 2023)
		FIV	Env	–	Critical for cell-cell fusion and Env incorporation into virions.	(González et al., 2012)
	Gammaretrovirus	MuLV	Env	–	Enhances lipid raft association, membrane stability, and virus assembly efficiency.	(Hensel et al., 1995; Li et al., 2002)
		MoMLV	Pr15E (R peptide)	–	Regulates fusion activity; may participate in virus budding.	(Olsen and Andersen, 1999)
	Deltaretrovirus	HTLV-1	p8/p12	–	Not essential for p8-mediated surface localization or virus transmission.	(Edwards et al., 2014)
	Alpharetrovirus	RSV	TM	–	Essential for protein stability, membrane localization, and virus infectivity.	(Ochsenbauer-Jambor et al., 2001)
Flaviviridae	Flavivirus	ZIKV	E	ZDHHC11	Negatively regulates viral infection.	(Hu et al., 2023)
		HCV	Core, NS2, NS4B	–	Regulates replication complex formation, virion assembly, and membrane association.	(Yu et al., 2006; Majeau et al., 2009; Paul et al., 2015; Wu et al., 2019)
		JEV	NS2A	–	Enhances protein stability, viral replication efficiency, and virulence.	(Ma et al., 2023)
Togaviridae	Alphavirus	SFV/Sindbis	nsP1, E1, E2, TF	–	Anchors replication complex to membranes; regulates budding and immune evasion (TF).	(Schmidt et al., 1988; Ivanova and Schlesinger, 1993; Laakkonen et al., 1996; Ahola et al., 2000; Smit et al., 2001; Karo-Astover et al., 2010; Ramsey et al., 2017; Ramsey et al., 2019; Rogers et al., 2020)
		CHIKV	nsP1, TF	ZDHHC2, 19	Targets nsP1 to cholesterol-rich microdomains for replication complex formation; regulates TF oligomerization and assembly.	(Yin et al., 2019; Zhang et al., 2019; Bakhache et al., 2020)
Herpesviridae	Simplexvirus	HSV-1/2	UL11, UL20, UL51	GODZ (DHHC3)	Regulates viral trafficking, envelope formation, and egress.	(Nozawa et al., 2003; Koshizuka et al., 2007; Shen et al., 2009; Wang et al., 2018)
	Cytomegalovirus	HCMV	gN, gB	–	Essential for secondary envelopment (gN) and membrane fusion (gB).	(Mach et al., 2007; Patrone et al., 2016)
	Lymphocryptovirus	EBV	LMP1	–	Mediates accumulation in signaling endosomes, driving oncogenicity.	(Verweij et al., 2015)
	Rhadinovirus	KSHV	pORF55	–	Critical for Golgi localization and efficient progeny virion production.	(Zhou et al., 2024)
Baculoviridae	Nucleopolyhedrovirus	AcMNPV	E26, GP64	–	Determines subcellular trafficking and functional switching (E26); non-essential for fusion/budding (GP64).	(Zhang et al., 2003; Burks et al., 2007)
Poxviridae	Orthopoxvirus	Vaccinia virus	F13L	–	Essential for membrane association and virus morphogenesis.	(Husain and Moss, 2003)
Adenoviridae	Mastadenovirus	Ad2/5	ADP	–	May regulate virus-induced cell lysis and virus release.	(Hausmann et al., 1998)
Hepeviridae	Orthohepevirus	HEV	ORF3	–	Essential for anchoring to membrane and secretion of quasi-enveloped virions.	(Gouttenoire et al., 2018; Liu et al., 2025)
Reoviridae	Orthoreovirus	ARV/NBV	p10	–	Essential for membrane fusion activity.	(Shmulevitz et al., 2003)
Arteriviridae	Porartevirus	PRRSV	N, GP5, M	ZDHHC3	Negatively regulates RNA synthesis but essential for virion assembly and budding.	(Zhang et al., 2021; Jing et al., 2025)
Asfarviridae	Asfivirus	ASFV	CP123L, QP383R	–	CP123L: promotes virion assembly and budding. QP383R: Inhibits the cGAS-STING innate immune response via cGAS S-palmitoylation.	(Hao et al., 2023; Guan et al., 2025)

This table summarizes the S-palmitoylated viral proteins discussed in this review. The “ZDHHCs” column lists only the palmitoyltransferases that have been explicitly reported in the cited literature. The “Major Functions & Mechanisms” column outlines the core roles and molecular mechanisms of this modification in key processes, including viral replication, assembly, and entry. “Refs.” corresponds to the citation numbers provided in the text. -: unknown.

### 3.1 Entry/fusion-associated proteins

The initial step of viral infection involves efficient attachment to and entry into host cells. Based on the presence or absence of an envelope, viruses are classified as enveloped and non-enveloped. These two types employ distinct entry mechanisms: enveloped viruses typically enter via receptor binding mediated by viral glycoproteins, followed by fusion of the viral envelope with the host cell membrane; non-enveloped viruses, by contrast, usually rely on endocytosis and subsequent penetration of the endosomal membrane.

#### 3.1.1 Enveloped viruses

The entry of enveloped viruses into host cells typically relies on two key steps mediated by their envelope glycoproteins: specific binding to host-cell surface receptors and subsequent membrane-fusion triggering. S-palmitoylation, a common post-translational modification, is found on the spike glycoprotein or envelope protein of various viruses. S-palmitoylation of these viral proteins typically occurs at cysteine residues within the cytoplasmic tail or proximal to the transmembrane domain. This modification facilitates their localization and anchoring to lipid rafts in the plasma membrane, ensures correct trafficking from the site of synthesis to the cell membrane, and enhances interactions with the membrane and matrix proteins to promote viral particle assembly. Furthermore, it regulates membrane fusion activity, thereby influencing viral entry and cell-to-cell spread. Importantly, functional differences exist among viruses and even subtypes; for instance, S-palmitoylation is not absolutely essential for the fusion activity of the H3 subtype influenza HA or the human respiratory syncytial virus (HRSV) F protein.

#### 3.1.2 Non-enveloped viruses

S-palmitoylation plays a crucial role in the membrane fusion process of specific non-enveloped viruses. As exemplified by the p10 protein of avian reovirus (ARV) and Nelson Bay reovirus (NBV), sequence alignment confirms the conservation of the membrane-proximal dicysteine motif (the palmitoylation site) in both p10 orthologs, and mutational analysis of this motif abolishes syncytium formation induced by either p10 protein (Shmulevitz et al., 2003). These examples highlight the conserved role of S-palmitoylation in promoting cell-cell fusion and dissemination among otherwise diverse viruses.

### 3.2 Replication-associated proteins

Viral genome replication-associated proteins are core components of viral replication, responsible for genome replication, transcription, or the assembly of replication complexes. S-palmitoylation of these proteins can anchor the replication complexes to cellular membranes and regulate protein-protein interactions within the replication machinery. For instance, in alphaviruses such as Semliki Forest virus (SFV) and Sindbis virus (SINV), the non-structural protein nsP1 is palmitoylated at cysteine residues in its C-terminal region (Cys418–420 in SFV and Cys420 in SINV). This modification enhances its binding to the plasma membrane, endosomes, and lysosomes, promotes localization to filopodia, and anchors the replication complex to the plasma membrane via an amphipathic helix. This modification is essential for formation and function of the replication complex (Laakkonen et al., 1996; Karo-Astover et al., 2010). Similarly, in Hepatitis C virus (HCV), S-palmitoylation at Cys172 of the core protein strengthens its association with the endoplasmic reticulum (ER) membrane, particularly the ER-adjacent lipid droplets. This modification promotes the assembly of viral particles on the ER membrane and facilitates their release via ER-derived transport and secretory pathways. Additionally, it induces lipid droplet accumulation, thereby maintaining a lipid environment conducive to viral replication. Mutation of Cys172 significantly reduces the formation of nucleocapsid-like particles (NLPs) in yeast cells and impairs viral particle assembly efficiency in Huh7.5 cells (Majeau et al., 2009). The PRRSV nucleocapsid (N) protein is one of the most abundant viral proteins in PRRSV-infected cells, and the formation of its homodimeric structure is essential for viral assembly and infectivity (Chen et al., 2019; Chang et al., 2020; Zheng et al., 2024). S-palmitoylation at the Cys90 residue of the N protein, catalyzed by ZDHHC3, exhibits a dual regulatory function: on the one hand, this modification interferes with the formation of the N protein-Nsp9 protein complex, inhibits viral RNA synthesis and thereby negatively regulates PRRSV replication (Jing et al., 2025); on the other hand, although the Cys90 mutant is capable of genomic replication and subgenomic mRNA transcription, it fails to produce infectious viral particles, confirming the necessity of this modification for viral assembly and maturation (Lee et al., 2005). Notably, this inhibitory effect of ZDHHC3 is counteracted by the APT1, which removes the palmitate group from N protein, thereby restoring N-Nsp9 interaction and promoting viral RNA synthesis. Pharmacological inhibition of APT1 with the small-molecule inhibitor ML348 effectively suppresses PRRSV-2 replication, highlighting the therapeutic potential of targeting depalmitoylation enzymes (Jing et al., 2025). This exemplifies the dynamic, reversible nature of S-palmitoylation in viral replication, where the balance between ZDHHC-mediated palmitoylation and APT1-mediated depalmitoylation determines the functional outcome. Furthermore, in both PRRSV-1 and PRRSV-2, the GP5 and M proteins are palmitoylated at conserved cysteine residues within their cytoplasmic tails near the transmembrane domain. This modification enables the fatty acid chains to interact with the lipid bilayer, driving the clustering of GP5/M heterodimers in cholesterol-enriched microdomains of the Golgi membrane and inducing membrane curvature, thereby promoting virus assembly and budding. Mutation of any or all S-palmitoylation sites significantly reduces virion production, but does not affect the formation of the disulfide-linked GP5-M dimer, ER-to-Golgi transport, or incorporation into virus particles, nor does it impair viral entry. However, these modifications are crucial for viral assembly and budding (Zhang et al., 2017; Zhang et al., 2021).

### 3.3 Assembly and release-associated proteins

Viral assembly and release represent the final sequential steps of the viral life cycle, involving the packaging of newly synthesized genomes and structural proteins into mature virions, followed by egress from host cells. S-palmitoylation of viral proteins involved in these processes primarily modulates membrane curvature (e.g., the amphipathic helix of M2 promotes budding), strengthens protein-protein interactions (e.g., S-palmitoylation of coronaviral E protein facilitates virion assembly), and mediates targeting to lipid rafts or specific cellular compartments (e.g., the Golgi apparatus and the plasma membrane). We classify these proteins into two distinct functional categories: structural proteins that focus on assembly and viroporins that serve dual purposes in ion balance and viral release.

#### 3.3.1 Structural proteins

The S-palmitoylation of N-terminal Cys9 in the UL51 protein of HSV-1 and duck virus enteritis (DEV) is critical for its Golgi targeting and membrane association, and may be involved in viral envelope assembly or egress (Nozawa et al., 2003; Shen et al., 2009). Similarly, the S-palmitoylation of N-terminal Cys11–13 in the HSV-2 UL11 protein localizes it to the plasma membrane and the Golgi apparatus and anchors it to lipid rafts, thereby promoting viral envelope formation and release. This raft association is thought to facilitate the recruitment of viral tegument and envelope components to specific membrane microdomains, which serve as platforms for secondary envelopment and budding (Koshizuka et al., 2007). Loss of S-palmitoylation results in the cytoplasmic retention of the protein and abolition of lipid raft-binding ability (Koshizuka et al., 2007).

#### 3.3.2 Viroporins

Viroporins are small, hydrophobic viral transmembrane proteins that self-oligomerize in membranes to form ion channels. Beyond modulating ion homeostasis to create a favorable replication environment, they often facilitate virus release by disrupting membrane integrity (Qu et al., 2025). S-palmitoylation of viroporins acts as a key regulatory switch that promotes virion assembly and release, rather than being essential for basal ion-channel activity. This modification functions by directing precise membrane targeting, stabilizing protein architecture, and facilitating membrane curvature and scission during budding. However, the functional necessity of this modification varies dramatically among different viroporins, reflecting distinct evolutionary adaptations.

Specifically, S-palmitoylation serves as a membrane anchor, which is a prerequisite for the plasma membrane localization of proteins like HEV ORF3 and the subsequent initiation of the viral secretion pathway (Gouttenoire et al., 2018; Liu et al., 2025). Concurrently, this modification ensures the integrity of virion assembly by stabilizing higher-order protein structures (e.g., the pentameric structure of the SARS-CoV-2 E protein) and preventing protein degradation (e.g., the MHV E protein) (Lopez et al., 2008; Sun et al., 2021; Wang et al., 2024). Functionally, the indispensability of S-palmitoylation is virus-specific. For HEV, MHV, and SARS-CoV-2, this modification is indispensable for the production or release of viral particles (Lopez et al., 2008; Gouttenoire et al., 2018; Wang et al., 2024). In contrast, for viroporins such as IAV M2 and ICV CM2, while S-palmitoylation is non-essential in cell culture, it fine-tunes viral fitness *in vivo*, reflecting functional optimization and redundancy (Holsinger et al., 1995; Grantham et al., 2009; Muraki et al., 2011). In summary, S-palmitoylation acts as a universal and critical molecular switch that governs the function of viroporins during the late stages of the viral life cycle.

### 3.4 Immune evasion proteins

To successfully establish infection, viruses employ host protein S-palmitoyltransferases to modify their own proteins or manipulate the S-palmitoylation of host proteins, thereby suppressing the host immune response. The following sections delineate the key strategies viruses have evolved to subvert host immunity through S-palmitoylation.

#### 3.4.1 Downregulation of MHC-I

Some viruses encode proteins that directly downregulate critical immune signaling molecules on the cell surface, and their function is often dependent on their own S-palmitoylation. A prime example is the Kaposi’s sarcoma-associated herpesvirus (KSHV) E3 ubiquitin ligase MIR2. MIR2 downregulates Major Histocompatibility Complex Class I (MHC-I) molecules from the surface of infected cells, enabling evasion from cytotoxic T lymphocytes (CTLs). This immune evasion function strictly requires MIR2 S-palmitoylation at Cys146 within its transmembrane domain. S-palmitoylation facilitates the interaction between MIR2 and the cytoplasmic tail of MHC-I, leading to K48-linked ubiquitination and subsequent proteasomal degradation of MHC-I. Mutation of Cys146 completely abrogates the ability of MIR2 to downregulate MHC-I, highlighting the critical role of this modification (Anania and Coscoy, 2011).

#### 3.4.2 Inhibition of cGAS-STING

A more direct strategy involves viruses actively manipulating the S-palmitoylation status of key host immune sensors to render them inactive. The African swine fever virus (ASFV) protein QP383R exemplifies this strategy. QP383R directly binds to the catalytic core of the cytosolic DNA sensor cGAS and recruits host palmitoyltransferases to promote cGAS S-palmitoylation. This virus-induced S-palmitoylation alters cGAS conformation, impairing its DNA-binding capacity and dimerization, thereby inhibiting the synthesis of the second messenger cGAMP and the subsequent generation of type I interferons through the cGAS-STING pathway (Zhou et al., 2018; He et al., 2022; Hao et al., 2023; Lu et al., 2023). Similarly, the Chikungunya virus (CHIKV) nonstructural protein nsP1 binds to STING, enhancing its S-palmitoylation and suppressing downstream signaling, effectively blocking DNA-induced interferon responses even in the context of an RNA virus infection (Webb et al., 2020). These cases clearly demonstrate that viruses can precisely exploit host palmitoyltransferases to directly silence central hubs of the antiviral defense system.

### 3.5 Concluding remarks on viral protein S-palmitoylation

In summary, S-palmitoylation, which serves as a versatile and often essential post-translational modification, is hijacked by viruses to facilitate multiple stages of their life cycle. From enhancing membrane fusion during entry (e.g., enveloped virus glycoproteins) to anchoring the replication complexes and promoting the assembly and release of viral particles, this reversible modification fine-tunes the function of viral protein function in a spatiotemporally regulated manner. Notably, S-palmitoylation can exert both positive and negative regulatory effects on viral replication, as exemplified by its role in PRRSV N protein. Additionally, viruses exploit this modification to directly undermine host immune surveillance, such as by downregulating MHC-I or inhibiting cGAS-STING signaling. Collectively, these findings underscore S-palmitoylation as a critical interface in virus-host interactions, highlighting its potential as a target for broad-spectrum antiviral interventions. The diverse strategies employed by different virus families are summarized in **Table 2**, illustrating how this modification is exploited across distinct lifecycle stages—from entry and replication to assembly and immune evasion.

**Table 2:** Comparative overview of S-palmitoylation and depalmitoylation strategies across virus families.

Virus family	Representative virus	Modified protein	Lifecycle stage	Host enzyme(s)	Functional consequences	Essentiality	Ref.
Coronaviridae	SARS-CoV-2	Spike (S) protein	Entry	ZDHHC20^Long^	Enhances viral infectivity and cell-cell fusion by promoting S protein palmitoylation at ERGIC; N-terminal extension of ZDHHC20^Long^ contains PERW motif for ER retention	Yes (for optimal infectivity)	(Mesquita et al., 2023)
		Envelope (E) protein	Assembly/Release	–	Stabilizes pentameric structure of viroporin; prevents protein degradation; facilitates virion assembly and release via membrane curvature induction	Yes (indispensable for particle production)	(Lopez et al., 2008; Sun et al., 2021; Wang et al., 2024)
	MHV	E protein	Assembly/Release	–	Palmitoylation prevents protein degradation; stabilizes viroporin structure	Yes (required for viral particle production)	(Lopez et al., 2008; Sun et al., 2021; Wang et al., 2024)
Orthomyxoviridae	Influenza A virus (IAV)	Hemagglutinin (HA)	Entry	DHHC1, 2, 4, 6 (for Flu B)	Promotes localization to lipid rafts; regulates membrane fusion activity; enhances interactions with matrix proteins	Subtype-dependent (non-essential for H3 subtype fusion activity)	(Meng and Veit, 2023)
		Matrix protein 2 (M2)	Assembly/Release	–	Amphipathic helix promotes membrane curvature and budding; S-palmitoylation fine-tunes viral fitness *in vivo*	Non-essential in cell culture; enhances fitness *in vivo*	(Holsinger et al., 1995; Grantham et al., 2009; Muraki et al., 2011)
Arteriviridae	PRRSV	Non-structural protein 1 (nsP1)	Replication	–	Anchors replication complex to plasma membrane via amphipathic helix; promotes localization to filopodia	Yes (essential for RC formation)	(Laakkonen et al., 1996; Karo-Astover et al., 2010)
		Nucleocapsid (N) protein	Replication	ZDHHC3 (palmitoylation)	Dual function: (1) Inhibits viral RNA synthesis by interfering with N-Nsp9 interaction; (2) Required for viral assembly and maturation	Yes (for infectious particle production)	(Lee et al., 2005; Jing et al., 2025)
				APT1 (depalmitoylation)	Removes palmitate from N protein; restores N-Nsp9 interaction; promotes viral RNA synthesis	Yes (essential for replication)	(Jing et al., 2025)
		Glycoprotein 5 (GP5) and Matrix (M) protein	Assembly	–	Drives clustering in cholesterol-rich Golgi microdomains; induces membrane curvature for budding	Yes (crucial for assembly/budding)	(Zhang et al., 2017; Zhang et al., 2021)
Togaviridae	Semliki Forest virus (SFV)	nsP1	Replication	–	C-terminal palmitoylation (Cys418-420) enhances plasma membrane, endosome, and lysosome binding	Yes (essential for RC function)	(Laakkonen et al., 1996)
	Sindbis virus (SINV)	nsP1	Replication	–	Palmitoylation at Cys420 anchors replication complex to plasma membrane	Yes (essential for RC formation)	(Karo-Astover et al., 2010)
	Chikungunya virus (CHIKV)	nsP1	Immune Evasion	–	Binds STING; enhances STING S-palmitoylation; suppresses downstream interferon signaling	Yes (blocks DNA-induced IFN responses)	(Webb et al., 2020)
Flaviviridae	Hepatitis C virus (HCV)	Core protein	Replication/Assembly	–	Cys172 palmitoylation strengthens ER membrane association; promotes assembly on ER and lipid droplets; induces lipid droplet accumulation	Yes (significantly reduces NLP formation when mutated)	(Majeau et al., 2009)
Herpesviridae	HSV-1	UL51 protein	Assembly	–	N-terminal Cys9 palmitoylation critical for Golgi targeting and membrane association; involved in envelope assembly/egress	Yes (required for proper localization)	(Nozawa et al., 2003; Shen et al., 2009)
	HSV-2	UL11 protein	Assembly	–	N-terminal Cys11–13 palmitoylation localizes to plasma membrane and Golgi; anchors to lipid rafts for secondary envelopment	Yes (loss causes cytoplasmic retention)	(Koshizuka et al., 2007)
	KSHV	MIR2 (E3 ligase)	Immune Evasion	–	Cys146 palmitoylation within transmembrane domain required for MHC-I downregulation; facilitates interaction with MHC-I cytoplasmic tail	Yes (mutation abrogates function)	(Anania and Coscoy, 2011)
Asfarviridae	ASFV	QP383R	Immune Evasion	Host palmitoyltransferases	Promotes cGAS S-palmitoylation; alters cGAS conformation; impairs DNA-binding and dimerization; inhibits cGAS-STING pathway	Yes (critical for immune evasion)	(Hao et al., 2023)
Hepeviridae	Hepatitis E virus (HEV)	ORF3 protein	Assembly/Release	–	S-palmitoylation serves as membrane anchor; prerequisite for plasma membrane localization and initiation of viral secretion pathway	Yes (indispensable for particle release)	(Gouttenoire et al., 2018; Liu et al., 2025)
Orthomyxoviridae	Influenza C virus (ICV)	CM2 protein	Assembly/Release	–	Viroporin palmitoylation fine-tunes viral fitness *in vivo*	Non-essential in cell culture; enhances fitness *in vivo*	(Holsinger et al., 1995; Grantham et al., 2009; Muraki et al., 2011)

This table summarizes how representative viruses from distinct families exploit S-palmitoylation and depalmitoylation at different lifecycle stages. The “Essentiality” column indicates whether the modification is absolutely required for the indicated function (Yes), dispensable *in vitro* but contributing to fitness *in vivo* (Context-dependent), or subtype-specific. Note that the same host depalmitoylase (e.g., APT1/LYPLA1) can exert opposing effects depending on whether its substrate is a viral or host protein, highlighting the complex interplay at the virus-host interface. Abbreviations: PRRSV, porcine reproductive and respiratory syndrome virus; HCV, hepatitis C virus; HSV, herpes simplex virus; KSHV, Kaposi’s sarcoma-associated herpesvirus; ASFV, African swine fever virus; HEV, hepatitis E virus; CHIKV, chikungunya virus; MHV, mouse hepatitis virus; IAV, influenza A virus; ICV, influenza C virus; ERGIC, ER-Golgi intermediate compartment; RC, replication complex; NLPs, nucleocapsid-like particles.

### 3.6 Viral hijacking of host palmitoyltransferase expression

Beyond targeting specific proteins, some viruses possess the capability to reprogram the host’s palmitoyltransferase system to enhance viral fitness. A prime example is SARS-CoV-2. Research has demonstrated that SARS-CoV-2 infection induces a shift in the transcriptional start site of the host ZDHHC20 gene. This shift triggers a switch from the expression of the canonical 42 kDa short isoform (ZDHHC20^short^) to a longer protein variant (ZDHHC20^Long^), which contains an additional 67-amino-acid extension at its N-terminus. Compared to ZDHHC20^short^, the ZDHHC20^Long^ isoform exhibits greater stability and a longer half-life. Its N-terminal extension harbors a PERW motif, which mediates its retention within the endoplasmic reticulum (ER). Notably, SARS-CoV-2 assembly occurs specifically at the ER-Golgi intermediate compartment (ERGIC). This relocalization allows ZDHHC20^Long^ to more efficiently palmitoylate the viral Spike protein, thereby enhancing viral infectivity and cell-cell fusion (Mesquita et al., 2023). Notably, this mechanism is not unique to the virus; it exploits an existing host damage-response pathway, as non-viral injuries can also trigger the expression of ZDHHC20^Long^ (Mesquita et al., 2023). It remains unknown whether ZDHHC20 exerts a similar mechanism in other viruses and whether its regulation exhibits viral specificity.

## 4 The role of DHHC enzymes in host antiviral innate immunity

S-palmitoylation, a dynamic and reversible lipid modification, acts as a pivotal regulatory node in orchestrating host innate antiviral responses. It precisely modulates the activation, amplification, and termination of immune signaling by regulating protein conformation, subcellular localization, and protein-protein interactions. This chapter focuses on four core antiviral defense systems characterized in the original literature: the cGAS-STING pathway (specialized in DNA virus recognition), the TRAF6-TAK1 pathway (mediating DNA virus-induced NF-κB activation), interferon-induced transmembrane (IFITM) proteins (broad-spectrum antiviral restriction factors) and MAVS (the mitochondrial antiviral-signaling protein). The regulatory roles of S-palmitoylation, including both catalytic (palmitoyltransferase activity-dependent) and non-catalytic (adaptor function-dependent, enzyme activity-independent) mechanisms, are elaborated in the following sections.

### 4.1 cGAS-STING

The cGAS-STING pathway is the primary innate immune signaling cascade for detecting cytosolic exogenous DNA (e.g., from DNA viruses such as HSV-1). S-palmitoylation modulates multiple steps of this pathway to balance antiviral efficacy and immune homeostasis.

#### 4.1.1 S-palmitoylation of STING

Upon DNA virus infection, viral DNA is released into the cytoplasm and recognized by cGAS. This activation of cGAS leads to the production of cyclic guanosine monophosphate-adenosine monophosphate (cGAMP), which binds to STING (also known as MITA, localized on the endoplasmic reticulum, ER) and triggers STING translocation to the Golgi apparatus and Golgi intermediate compartment (Dvorkin et al., 2024). A critical step in STING activation is its S-palmitoylation at Cys88/Cys91, catalyzed by palmitoyltransferases DHHC3, DHHC7, and DHHC15 (Mukai et al., 2016). This modification traps STING in the trans-Golgi network (TGN) to prevent premature degradation, promotes STING oligomerization and colocalization with lipid rafts, and facilitates the recruitment of TANK-binding kinase 1 (TBK1) and interferon regulatory factor 3 (IRF3) to form a STING-TBK1-IRF3 signalosome, ultimately inducing IRF3 phosphorylation and transcription of type I interferons (e.g., IFN-I, IFN-β) (Mukai et al., 2016). Loss of STING S-palmitoylation (e.g., via Cys88/Cys91 mutation) abrogates IFN-I production, highlighting the indispensability of this modification for cGAS-STING pathway activation.

#### 4.1.2 Regulation by ZDHHCs

Multiple ZDHHC proteins fine-tune the cGAS-STING pathway through either catalytic or non-catalytic mechanisms:

##### 4.1.2.1 Positive regulators by non-catalytic functions

ZDHHC1: Acts as an adaptor protein (independent of its palmitoyltransferase activity) by binding to STING (known as MITA) via its N-terminal transmembrane domain. This interaction enhances STING dimerization/aggregation, promotes the assembly of the STING-TBK1-IRF3 complex, and upregulates the expression of IFN-I and proinflammatory cytokines (Zhou et al., 2014). ZDHHC1-knockout (KO) cells exhibit reduced cytokine production in response to DNA viruses (e.g., HSV-1) but no response to RNA viruses (e.g., SeV, VSV); ZDHHC1 KO mice infected with HSV-1 show lower serum cytokines, higher brain viral titers, and higher mortality (Zhou et al., 2014). Notably, the antiviral role of ZDHHC1 in mandarin fish (Siniperca chuatsi) is consistent with its characterized function in mammals (Chen et al., 2022).

ZDHHC11: Similarly functions independently of palmitoyltransferase activity. It interacts directly with STING (via STING’s N-terminal transmembrane domain, aa 1-190) and IRF3 (via ZDHHC11’s C-terminal region, aa 198-412), specifically mediating IRF3 recruitment to STING (Liu et al., 2018). Overexpression of ZDHHC11 activates the IFN-β promoter and NF-κB, while ZDHHC11 deficiency inhibits HSV-1-induced IFNB1/IL6 expression and IRF3 phosphorylation (without affecting TBK1 phosphorylation). Importantly, ZDHHC11 KO mice infected with HSV-1 display decreased serum IFN-α, IFN-β, TNF-α, and IL-6, elevated brain viral loads, and higher mortality—with no significant impact on RNA virus (SeV, EMCV) infections (Liu et al., 2018).

Collectively, the shared characteristics of ZDHHC1 and ZDHHC11 highlight the features that underlie their function as non-catalytic regulators: 1) Phylogenetic analysis of the human ZDHHC proteins reveals that ZDHHC1 is closely related to ZDHHC11 (Korycka et al., 2012). 2) The localization of ZDHHC1 and ZDHHC11 on the endoplasmic reticulum (De and Sadhukhan, 2018; Liu et al., 2021) enables their constitutive interaction with STING at the ER membrane.

##### 4.1.2.2 Negative regulator (catalytic function)

ZDHHC18 acts as a negative regulator by catalytically palmitoylating cGAS at Cys474. This modification alters cGAS conformation, impairs its binding to double-stranded DNA (dsDNA), and suppresses cGAS dimerization—thus dampening cGAS-mediated IFN-I production and immune defense against DNA viruses (Shi et al., 2022). Consistent with this, ZDHHC18 KO cells and mice exhibit enhanced resistance to DNA virus infection, confirming the role of ZDHHC18 as a negative checkpoint (Shi et al., 2022).

### 4.2 TRAF6-TAK1

The tumor necrosis factor receptor-associated factor 6 (TRAF6)-transforming growth factor-β-activated kinase 1 (TAK1) pathway is critical for activating the NF-κB and MAPK signaling cascades, which regulate the production of proinflammatory cytokines during antiviral immunity. Although ZDHHC11 can mediate S-palmitoylation, its regulation of the TRAF6-TAK1 pathway in response to DNA viruses is independent of this enzymatic activity. Instead, ZDHHC11 functions in a non-catalytic adaptor capacity.

The C-terminal region (amino acids 198-412) of ZDHHC11 binds to the TRAF-C domain of TRAF6, promoting TRAF6 oligomerization (Liu et al., 2021). Enhanced TRAF6 oligomerization significantly increases its E3 ubiquitin ligase activity, facilitating the synthesis of K63-linked polyubiquitin chains. These chains activate TAK1 and the IκB kinase (IKK) complex, leading to activation of NF-κB pathway and transcription of proinflammatory cytokines (e.g., IL-6, TNF-α) (Liu et al., 2021). It is worth noting that this regulatory effect is independent of the palmitoyltransferase activity of ZDHHC11-mutation of its DHHC domain (the catalytic core) does not affect the activation of NF-κB (Liu et al., 2021).

ZDHHC11 KO bone marrow-derived macrophages (BMDMs) and mouse embryonic fibroblasts (MEFs) show reduced NF-κB activation induced by IL-1β, LPS, or HSV-1 (DNA virus), and decreased mRNA levels of downstream proinflammatory cytokines (IL-6, TNF-α). No such effect is observed for RNA viruses (e.g., SeV) (Liu et al., 2021). ZDHHC11 KO mice that are infected with HSV-1 show markedly lower levels of serum IL-6 and weakened antiviral immune responses (Liu et al., 2021).

### 4.3 IFITMs

Interferon-induced transmembrane (IFITM) proteins are evolutionarily conserved broad-spectrum antiviral restriction factors. The vertebrate IFITM family is divided into three subfamilies: immune-related IFITMs (IFITM1, IFITM2, IFITM3, IFITM6, IFITM7), IFITM5, and IFITM10. Among these, only IFITM1, IFITM2, and particularly IFITM3 exhibit antiviral activity (Gómez-Herranz et al., 2023). Notably, S-palmitoylation is one of the key mechanisms regulating the antiviral activities of these three proteins.

#### 4.3.1 IFITM1

The antiviral activity of IFITM1 (e.g., against Japanese encephalitis virus, JEV) depends on S-palmitoylation of conserved cysteine residues within its CD225 domain.

The human protein IFITM1 (hIFITM1) is anchored to the endothelial cell membrane of the blood-brain barrier (BBB) through palmitoylation at conserved cysteine residues 51, 52, and 84 in its CD225 domain, which prevents its cytoplasmic retention. This post-translational modification is essential for hIFITM1 to exert dual biological functions: first, palmitoylated hIFITM1 directly interacts with the tight junction protein Occludin at the cell membrane, which strengthens the endothelial monolayer integrity, elevates transendothelial electrical resistance (TEER), and reduces paracellular permeability to macromolecules and viral particles; second, membrane-localized hIFITM1 blocks the fusion process between the Japanese encephalitis virus (JEV) envelope and host cell membrane, thereby inhibiting viral entry and replication in human brain microvascular endothelial cells (hBMECs). In contrast, mutation of these palmitoylation sites traps hIFITM1 in the cytoplasm, impairs its binding to Occludin, disrupts BBB homeostasis, and abolishes its antiviral activity against JEV (Chen et al., 2024).

In swine IFITM1 (sIFITM1), S-palmitoylation of cysteine residues 50, 51, and 84 is critical for its anti-JEV activity. Specifically, palmitoylation at Cys84 is essential for maintaining proper subcellular localization and protein stability. Loss of palmitoylation at C84 (C84S mutation) results in perinuclear aggregation, accelerated proteasomal degradation, and complete abolishment of antiviral function (Xu et al., 2020). This suggests that S-palmitoylation anchors sIFITM1 to specific membrane compartments (likely early endosomes and plasma membrane), where it restricts viral entry by preventing fusion or promoting endolysosomal trafficking of JEV virions.

Similarly, murine IFITM1 (mIFITM1) undergoes S-palmitoylation at three conserved cysteines (Cys49, Cys50, Cys83) and one non-conserved cysteine (Cys103). This modification, catalyzed by palmitoyltransferase ZDHHC7, serves dual functions: 1) preventing proteasomal degradation by enhancing protein stability, and 2) promoting proper intramembrane topology with cytoplasm-oriented termini, which is essential for restricting influenza A virus (IAV) entry (Hach et al., 2013). The C-terminal palmitoylation at C103 further supports membrane insertion and antiviral activity when conserved sites are compromised.

Viruses (e.g., JEV) counteract this defense by disrupting IFITM1 S-palmitoylation: JEV infection downregulates palmitoyltransferases ZDHHC1, ZDHHC23, and ZDHHC24, while upregulating the depalmitoylase ABHD16A—collectively reducing hIFITM1 S-palmitoylation and antiviral potency (Chen et al., 2024; Wen et al., 2025).

#### 4.3.2 IFITM3

S-palmitoylation is a key post-translational modification that regulates the antiviral activity of IFITM3, and its modification mechanism, functional impact, and species-specific features have been extensively elucidated. Studies have shown that S-palmitoylation of human and murine IFITM3 mainly occurs at three cysteine residues: Cys71, Cys72, and Cys105, among which Cys72 is the most critical site, essential for maintaining its antiviral function (Yount et al., 2010; Garst et al., 2021). This modification is catalyzed by multiple members of the ZDHHC enzyme family, such as ZDHHC3, 7, 15, and 20, which exhibit functional redundancy. Consequently, the loss of any single member does not significantly impair IFITM3 S-palmitoylation or its antiviral function (McMichael et al., 2017). Notably, ZDHHC20 displays a unique advantage: it extensively co-localizes with IFITM3 in lysosomes, whereas other ZDHHC members are localized in the perinuclear region. This specific localization enables ZDHHC20 to significantly enhance the inhibitory effect of IFITM3 on influenza virus when overexpressed, and this enhancing effect depends on its catalytic activity (McMichael et al., 2017).

The IFITM3 function regulated by S-palmitoylation is manifested at multiple levels. On one hand, it promotes the clustering of IFITM3 within membrane structures, strengthens its binding to lipid membranes, and thereby influences the fusion process between the virus and the endosomal membrane (Yount et al., 2010). On the other hand, palmitoylated IFITM3 can inhibit viral entry into the cytoplasm either by accelerating the trafficking and clearance of viral particles to lysosomes or by altering the rigidity and curvature of the endosomal membrane (Garst et al., 2021). From a species-specific perspective, bat IFITM3 also relies on S-palmitoylation at Cys71, Cys72, and Cys105 for its antiviral function. Mutation of any of these sites impairs its ability to restrict viruses such as Zika and influenza viruses, while the triple mutant completely loses antiviral activity, accompanied by a shift in subcellular localization toward the Golgi apparatus (Benfield et al., 2020). Furthermore, a polymorphism at Codon70 within the CD225 domain of bat IFITM3 indirectly affects the efficiency of S-palmitoylation, thereby modulating its antiviral activity. In contrast, mutation at the Codon70 site in human IFITM3 does not significantly impact its activity (Benfield et al., 2020).

Further structural biology studies have revealed that S-palmitoylation at Cys72 induces a conformational change in the amphipathic helix 1 (AH1) of IFITM3, enabling it to anchor more tightly to the lipid membrane. This conformational stabilization serves as the structural basis for its enhanced antiviral activity (Garst et al., 2021). Furthermore, S-palmitoylation, along with other post-translational modifications such as ubiquitination and methylation, forms a regulatory netword for IFITM3. Among these, S-palmitoylation is the only known positive regulatory modification, while others often exert negative regulatory effects by influencing its localization or stability (Yount et al., 2010; McMichael et al., 2017).

These findings not only clarify the central role of S-palmitoylation in the antiviral mechanism of IFITM3 but also provide a theoretical basis for developing broad-spectrum antiviral strategies by targeting the regulation of IFITM3 S-palmitoylation levels.

### 4.4 MAVS

In the innate immune defense against viral infections, the retinoic acid-inducible gene I (RIG-I)-like receptor (RLR) signaling pathway serves as a critical mechanism for recognizing RNA viruses and initiating antiviral responses. Upon RNA virus infection, RLRs detect viral nucleic acids and undergo conformational changes. Through their caspase activation and recruitment domains (CARD), they interact with the CARD domain of MAVS, inducing MAVS to form prion-like aggregates (Chen et al., 2021). These aggregates recruit downstream kinases such as TANK-binding kinase 1 (TBK1) and the IκB kinase (IKK) complex, leading to the activation of transcription factors NF-κB and IRF3/7. This cascade ultimately induces the expression of type I interferons (e.g., IFN-β), proinflammatory cytokines, and interferon-stimulated genes (ISGs), establishing an antiviral state in the host (Sharma et al., 2021).

As the central adaptor protein in the RLR pathway, MAVS function is tightly regulated by various post-translational modifications and protein-protein interactions. Recent studies have revealed that S-palmitoylation of MAVS plays a crucial regulatory role in its mediated antiviral immune response. Different members of the ZDHHC family of palmitoyltransferases have been shown to modulate MAVS function through distinct mechanisms, highlighting the complexity and precision of this regulatory layer in antiviral innate immunity.

S-palmitoylation of MAVS is a key regulatory mechanism in the antiviral innate immune response, and its modification sites, catalytic enzymes, and functional effects have been gradually elucidated through multiple studies. Research has confirmed that S-palmitoylation of MAVS can occur at multiple conserved cysteine residues. Among these, C79 is the first identified core site, and its S-palmitoylation is primarily catalyzed by ZDHHC12. This modification is essential for MAVS to form prion-like aggregates and activate the downstream type I interferon (IFN) pathway (Wang et al., 2024). ZDHHC12 interacts with the full-length MAVS and enhances its S-palmitoylation level upon RNA virus infection. The systemic lupus erythematosus-associated MAVS mutant C79F, which lacks this modification site, impairs IFN production and increases host susceptibility to viral infection (Wang et al., 2024).

Another study has demonstrated that MAVS residue C508 (adjacent to its C-terminal transmembrane domain) can be palmitoylated by ZDHHC7. This modification does not affect the mitochondrial localization of MAVS under resting conditions but significantly enhances the stability of MAVS aggregates on the outer mitochondrial membrane during viral infection, promoting IRF3 phosphorylation and IFN secretion (Liu et al., 2024). ZDHHC3 also exhibits weak catalytic activity. Deficiency of ZDHHC7 markedly reduces antiviral immune responses in macrophages and mice, increasing the replication of influenza virus and encephalomyocarditis virus. The C508S mutant forms only dispersed small punctate aggregates upon stimulation and partially detaches from mitochondria, indicating that S-palmitoylation regulates immune signaling by enhancing the membrane-binding capacity and aggregate stability of MAVS (Liu et al., 2024). This study complements the mechanism of C79 site modification, together revealing the fine-tuning of MAVS aggregation by S-palmitoylation at different stages.

Recent research has further identified C46 and C79 of MAVS as S-palmitoylation sites, catalyzed by ZDHHC24. Palmitic acid can promote this modification and the mitochondrial localization and aggregation of MAVS by enhancing the interaction between ZDHHC24 and MAVS (Bu et al., 2024). Deficiency of ZDHHC24 significantly impairs the activation of the TBK1-IRF3 pathway and IFN production induced by RNA viruses, while a high-palmitic acid diet can enhance antiviral immunity in mice in a ZDHHC24-dependent manner (Bu et al., 2024). Additionally, depalmitoylase APT2 can specifically remove S-palmitoylation from MAVS. The inhibitor ML349 significantly enhances the formation of MAVS aggregate and antiviral responses by blocking this process, offering a potential strategy for targeting the regulation of MAVS S-palmitoylation (Bu et al., 2024).

In summary, S-palmitoylation of MAVS at distinct sites (C46, C79, C508) and its coordinated modification by specific ZDHHC family members (ZDHHC7, ZDHHC12, ZDHHC24) regulate its aggregate stability, mitochondrial localization, and downstream signaling activation. This constitutes a critical regulatory network in antiviral innate immunity and provides novel molecular targets for the treatment of related diseases, such as autoimmune disorders and viral infections.

### 4.5 Concluding remarks on S-palmitoylation in host antiviral immunity

Overall, S-palmitoylation serves as a pivotal regulatory mechanism across multiple arms of the host antiviral innate immune response. Through site-specific modifications catalyzed by distinct ZDHHC enzymes, it governs the membrane association, stability, aggregation, and signaling competency of key immune adaptors and effectors, including STING, MAVS, and IFITMs. While the modification enhances immune activation of pathways such as cGAS-STING and RLR-MAVS, it also fine-tunes the antiviral efficacy of restriction factors like IFITM3. Notably, the regulatory roles of ZDHHC proteins extend beyond catalytic activity, as exemplified by the scaffolding functions of ZDHHC1 and ZDHHC11 in promoting signalosome assembly. These findings underscore a sophisticated layer of post-translational control that enables the host to launch rapid and tailored defenses against viral invasions. Importantly, this host-centric regulation stands in dynamic contrast to the previously described viral hijacking of S-palmitoylation mechanisms, highlighting the constant tug-of-war at the virus-host interface. The reversibility of this modification further implies that the net immune outcome is determined by the balance between palmitoylation and its counterpart—depalmitoylation, which we will discuss in the following section.

## 5 The role of depalmitoylation in viral infection and host immunity

As mentioned previously, the appearance of viral disease symptoms is a result of both direct damage by the virus and the host’s immunopathological responses (Freer and Matteucci, 2009). However, the regulatory effects of depalmitoylation in these processes have only recently begun to emerge. Notably, depalmitoylases can exert dichotomous effects—either promoting or restricting viral infection depending on substrate identity and cellular context (**Table 3**). Emerging evidence clearly indicates that depalmitoylation is crucial for the invasion and replication of RNA viruses, as well as for their interaction with the host innate immune response. So far, only four depalmitoylases, namely APT1, APT2, PPT1, and ABHD16A, have been identified as participants in these processes, underscoring their potential value as core regulatory nodes.

**Table 3:** Context-dependent functions of host depalmitoylases in virus-host interactions.

Depalmitoylase	Virus	Target	Functional Outcome	Ref.
APT1	PRRSV	Viral N protein	Pro-viral: Removes palmitate to restore N-Nsp9 interaction; promotes RNA synthesis	Jing et al., 2025
	FMDV	–	Pro-viral: Upregulated by infection; knockdown reduces viral titers	Guo et al., 2015
	VSV, IAV, SFV	Viral glycoproteins (G, HA, E2)	In vitro antiviral: Removes palmitic acid from viral envelope proteins	Veit and Schmidt, 2001
	SARS-CoV-2	Host ACE2 receptor	Antiviral: Depalmitoylation reduces ACE2 surface stability; limits viral entry	Xie et al., 2001
APT2	RNA viruses (VSV, SeV)	Host MAVS	Immune regulation: Removes palmitate from MAVS; compromises aggregation and signaling; inhibitor ML349 enhances antiviral immunity	Bu et al., 2024

This table illustrates the dichotomous roles of depalmitoylases, which can be hijacked by viruses to facilitate replication (pro-viral) or act as intrinsic restriction factors limiting viral entry (antiviral). The same enzyme (e.g., APT1/LYPLA1) may exert opposing effects depending on substrate identity—viral versus host proteins—highlighting the pleiotropic nature of palmitoylation dynamics at the virus–host interface. Pharmacological modulation of these enzymes (e.g., ML348 for APT1, ML349 for APT2) represents a potential therapeutic strategy, though the directionality of intervention (inhibition versus activation) must be carefully evaluated on a case-by-case basis.

### 5.1 Depalmitoylation in viral replication

Depalmitoylases play a multifaceted role in the replication of viruses, functioning as a double-edged sword that may either promote or hinder the spread of viral entities.

Early *in vitro* studies by Veit et al. have demonstrated that the depalmitoylase APT1 could remove palmitic acid from the glycoproteins of several enveloped RNA viruses, including the G protein of vesicular stomatitis virus (VSV), the hemagglutinin of influenza viruses, and the E2 glycoprotein of Semliki Forest virus (SFV). Interestingly, APT1 exhibited poor activity toward the SFV E1 glycoprotein, hinting at an early recognition of substrate specificity among viral proteins (Veit and Schmidt, 2001). This differential susceptibility may stem from distinct local conformations or steric hindrance at the cytoplasmic tail-membrane interface of E1, or it may be attributable to the difference in the number of acylation sites (E1 harbors a single site versus four in E2). In contrast to these early *in vitro* findings, subsequent cellular studies revealed that some viruses actively exploit host depalmitoylases to enhance their replication. For instance, infection with foot-and-mouth disease virus (FMDV) upregulated the expression of the depalmitoylase APT1, and knockdown of APT1 significantly reduced the synthesis and titers of RNA viruses, indicating a pro-viral role (Guo et al., 2015). A more direct mechanism was observed in Porcine Reproductive and Respiratory Syndrome Virus (PRRSV), where APT1-mediated depalmitoylation of the viral nucleocapsid (N) protein reversed the inhibitory effect of ZDHHC3-mediated S-palmitoylation, restoring the interaction between the N protein and Nsp9 and facilitating the synthesis of viral RNA (Jing et al., 2025). The small-molecule inhibitor ML348, which inhibits APT1, significantly diminishes PRRSV replication, highlighting the potential for therapeutic strategies targeting this enzyme (Jing et al., 2025).

Conversely, depalmitoylases can also act as antiviral factors by modifying host proteins required for viral entry. A prime example is the SARS-CoV-2 receptor, angiotensin-converting enzyme 2 (ACE2). Knockdown of APT1 increases the S-palmitoylation of ACE2, leading to enhanced stabilization on the plasma membrane. This elevated surface expression of ACE2 consequently increases cellular susceptibility to SARS-CoV-2 infection. Accordingly, pharmacological inhibition of APT1 with ML348 boosts SARS-CoV-2 infection efficiency (Xie et al., 2021). This presents a scenario where the same depalmitoylase (APT1) can be pro-viral in one context (e.g., PRRSV) and anti-viral in another (e.g., SARS-CoV-2), depending on whether its substrate is a viral or a host protein.

Collectively, these findings reveal that the influence of depalmitoylases on viral replication is not symmetric. They can be hijacked by viruses to promote their own life cycle through the depalmitoylation of viral proteins, or they can serve as innate barriers by modulating the localization and stability of critical host factors like viral receptors. What, then, determines whether a depalmitoylase acts as a proviral facilitator or an antiviral restriction factor? First, substrate identity is paramount: depalmitoylation of viral proteins (e.g., PRRSV N protein) typically benefits viral replication by reversing host ZDHHC-mediated restriction, whereas depalmitoylation of host proteins required for viral entry, such as the SARS-CoV-2 receptor ACE2, limits infection by reducing receptor surface availability. Second, spatiotemporal expression patterns during infection play a critical role; viruses like FMDV actively upregulate APT1 expression early in infection to enhance replication, whereas in other contexts, constitutive depalmitoylase activity may constitutively suppress viral spread. Third, subcellular compartmentalization determines substrate accessibility; depalmitoylases localized to viral replication factories (e.g., ER-derived membranes) likely encounter viral substrates preferentially, while those at the plasma membrane may predominantly target host entry factors. Fourth, the dynamic balance between ZDHHC-mediated palmitoylation and depalmitoylase activity creates a rheostat-like effect; the net palmitoylation status depends on the relative enzymatic activities, which may vary across cell types, metabolic states, or infection stages. Finally, cell-type-specific expression profiles of depalmitoylase isoforms (e.g., neuron-enriched PPT1 versus ubiquitously expressed APT1/LYPLA1) likely dictate tissue tropism and viral pathogenesis. Understanding these determinants will be essential for predicting when pharmacological inhibition versus enhancement of depalmitoylase activity would constitute an effective antiviral strategy.

### 5.2 Depalmitoylation in innate immunity

Beyond their direct roles in modulating viral proteins and host receptors, depalmitoylases also play a critical role in fine-tuning the host innate immune response. This regulatory function ensures a robust yet controlled antiviral defense, thereby preventing immunopathology. The following examples illustrate how depalmitoylation acts as a key regulator of major innate immune signaling pathways.

The mitochondrial antiviral-signaling protein (MAVS) serves as a crucial regulatory component within the RLR pathway, facilitating the production of type I interferon in response to RNA virus detection. Recent research has identified depalmitoylation as a critical negative feedback mechanism for MAVS. The depalmitoylase APT2 specifically removes palmitoyl groups from MAVS via thioester bond hydrolysis. This depalmitoylation compromises the aggregation and mitochondrial localization of MAVS, which are essential for its signal transduction function. Consequently, APT2 activity results in reduced phosphorylation of TBK1 and IRF3, decreased interferon production, and an attenuated antiviral state. The significance of this regulation is underscored by the fact that the APT2 inhibitor ML349 stabilizes palmitoylated MAVS, restoring its activation and significantly enhancing antiviral immunity against viruses like VSV and SeV both *in vitro* and *in vivo* (Bu et al., 2024).

Interestingly, depalmitoylases can also utilize non-enzymatic mechanisms to bolster antiviral defense. Conventional dendritic cells type 1 (cDC1), which is a professional antigen-presenting cell vital for initiating adaptive immunity, employs the depalmitoylase PPT1 in a unique manner. In cDC1s, PPT1 is recruited to phagosomes, where it plays a critical role in maintaining an acidic environment required for viral inactivation, presumably by scaffolding and recruiting the V-ATPase proton pump complex. This function may be unrelated to its canonical depalmitoylase activity (Ou et al., 2019). Furthermore, PPT1 expression is under dynamic regulation: it is highly expressed at steady state to sustain this phagosomal antiviral function, but is rapidly downregulated upon cDC1 activation to facilitate antigen retention and cross-presentation, thereby perfectly balancing innate viral clearance with adaptive immune activation (Ou et al., 2019).

In summary, depalmitoylases serve as precise rheostats for innate immunity. Through enzymatic activity, as seen with APT2 fine-tuning MAVS, they prevent excessive immune activation. Additionally, through both enzymatic and non-enzymatic support roles, as demonstrated by PPT1 in cDC1s, they play a crucial role in enhancing the cell’s defenses against viral threats.

### 5.3 Concluding remarks on depalmitoylation in virus-host interactions

Collectively, the studies discussed herein suggest that depalmitoylation is not merely a passive reversal of S−palmitoylation but an active regulatory layer that shapes viral infection and host immunity. Depalmitoylases function as a double−edged sword: they can be co−opted by viruses to promote replication (e.g., APT1 in PRRSV) or act as host restriction factors by modulating critical proteins such as ACE2 or MAVS. Moreover, their roles extend beyond canonical enzymatic activity, as exemplified by PPT1’s non−catalytic scaffolding function in dendritic cells. Despite these advances, only a subset of known depalmitoylases (APT1, APT2, PPT1, ABHD16A) has been firmly linked to virus-host interactions, leaving a vast landscape for future discoveries.

## 6 Conclusions

This review expounds upon the current understanding of S-palmitoylation and depalmitoylation, highlighting their pivotal regulatory roles at the virus-host interface. These reversible modifications orchestrate multiple stages of the viral lifecycle (entry, replication, assembly, and egress), while mediating the interplay between host innate immune activation and viral evasion strategies. As obligate intracellular parasites, viruses hijack the host S-palmitoylation machinery to enhance their fitness and subvert antiviral defenses, whereas the host counteracts by leveraging this system to potentiate innate immune signaling and antiviral restriction factors.

**Figure 3:**
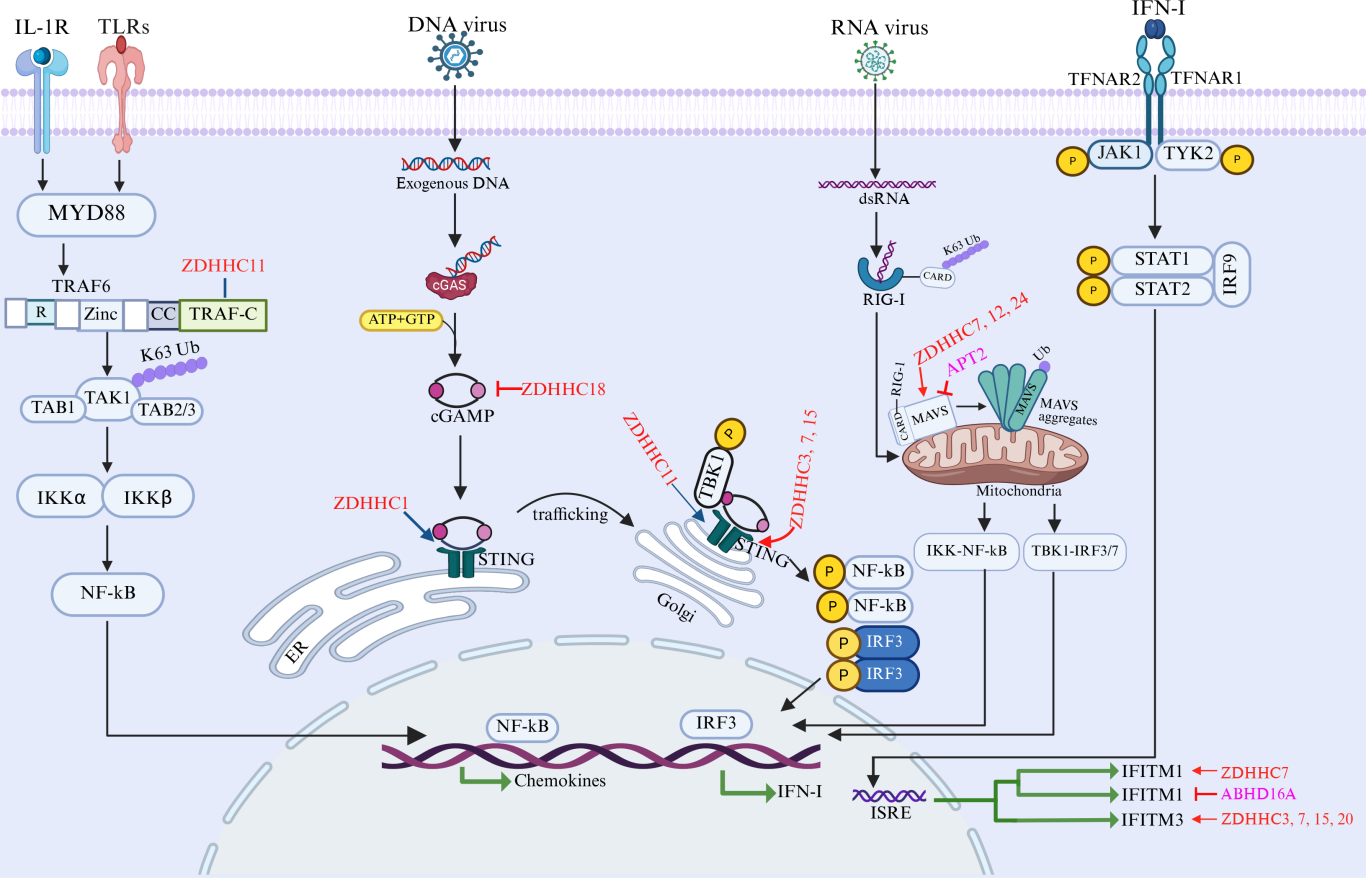
The role of S-Palmitoylation and depalmitoylation in antiviral innate immune signaling. Cell membrane-localized TLR/IL-1R receptors can activate NF-κB via MYD88. In the cytosol, cGAS recognizes foreign double-stranded DNA and catalyzes the synthesis of the second messenger cGAMP from ATP and GTP. cGAMP then binds to and activates the endoplasmic reticulum (ER) resident protein STING. This binding triggers the translocation of STING from the ER to the Golgi apparatus, where it recruits TBK1. TBK1 subsequently phosphorylates and activates the transcription factors IRF3 and NF-κB. Their activation ultimately induces the production of type I interferons (IFN-I) and pro-inflammatory cytokines. Secreted IFN-I binds to the IFNAR receptor, initiating the JAK-STAT signaling pathway. This leads to the expression of interferon-induced transmembrane (IFITM) proteins and the establishment of an antiviral state in the cell. Mitochondrial antiviral-signaling protein (MAVS) protein, as an adaptor molecule, the MAVS detects signals from viral RNA sensors such as RIG-I and MDA5 through its CARD domain. MAVS subsequently aggregates and recruits downstream proteins including TRAFs, TBK1, and IKK, thereby inducing anti-viral immune response. APT2 and ABHD16A, which catalyze the depalmitoylation of MAVS and IFITM1, respectively. P, phosphate; Ub: ubiquitin; ER, endoplasmic reticulum; ISRE, IFN-stimulated response element; dsRNA, double-stranded RNA; **⊢**, negative regulation. Created in BioRender. ruiqin, X. (2026) https://BioRender.com/1buzbzv is licensed under CC BY 4.0.

A comprehensive schematic summarizing these intricate interactions is presented in **Figure 3**. As depicted, the functional outcome of this interplay is a finely tuned balance determined by the concerted actions of ZDHHC palmitoyltransferases and depalmitoylases on both viral and host proteins. Notably, the regulatory mechanisms transcend canonical enzymatic activity. Certain ZDHHC palmitoyltransferases (e.g., ZDHHC1 and ZDHHC11) and depalmitoylases (e.g., PPT1) exert critical functions through non-catalytic, scaffolding roles, adding a significant layer of complexity to this regulatory network.

Despite these advances, major gaps persist in linking specific depalmitoylases to viral substrates and understanding their catalytic versus scaffolding functions in infection. Experimentally, while reverse genetics has largely defined the S-palmitoylation of individual viral proteins, systematic identification of depalmitoylases acting on viral substrates remains in its infancy. Future efforts should combine palmitoyl-proteomics (palmitoylomics) in infected cells with CRISPR-Cas9 screens targeting the entire repertoire of known depalmitoylases, including the APT1/2, PPT1, and ABHD17 family members, to systematically map their virus-specific substrates and spatiotemporal dynamics during infection. Furthermore, integrating proximity labeling techniques (such as BioID or APEX) with quantitative mass spectrometry could capture transient enzyme-substrate interactions that conventional co-immunoprecipitation might miss, particularly for dissecting non-catalytic scaffolding functions. Structural biology approaches, including cryo-EM and AlphaFold-multimer predictions, should be employed to resolve the molecular interfaces governing substrate recognition, thereby facilitating the rational design of substrate-specific inhibitors. Additionally, time-resolved palmitoylomic analyses across distinct stages of the viral lifecycle (entry, replication, egress) will be essential to unravel how the balance between palmitoylation and depalmitoylation is temporally orchestrated to regulate viral fitness and immune evasion. Integrating these multi-omic approaches with chemical biology tools (Puthenveetil et al., 2023; Ocasio et al., 2024) will be crucial to unravel the specificity of this reversible modification and map the entire regulatory network.

Targeting the reversible palmitoylation machinery represents a promising yet challenging antiviral strategy. As obligate intracellular parasites, viruses rely on host S-palmitoylation enzymes throughout their life cycle. Targeting host factors raises the genetic barrier to viral resistance, as viruses cannot easily overcome their dependence on specific host cellular pathways through self-mutation—a key theoretical advantage of host-directed strategies over direct-acting antivirals. However, several major hurdles must be overcome. First, the mammalian ZDHHC family comprises 23 members with partially overlapping substrate spectra, and many host proteins essential for cell viability are also palmitoylation-dependent. Broad-spectrum inhibition of ZDHHCs or depalmitoylases may disrupt critical host signaling pathways, leading to unacceptable cytotoxicity. Second, the high conservation of the catalytic domains of palmitoyltransferases complicates the development of isoform-specific small-molecule inhibitors. Third, tissue-specific toxicity poses a significant risk, particularly for the nervous system—deficiency of the depalmitoylase PPT1 causes infantile neuronal ceroid lipofuscinosis-1 (CLN1 disease), underscoring the essential role of palmitoylation homeostasis in neuronal survival.

To address these challenges, future therapeutic strategies should prioritize: (1) targeting protein-protein interaction interfaces rather than catalytic sites to achieve substrate-specific blockade; (2) exploiting the non-catalytic scaffolding functions of specific ZDHHCs (e.g., ZDHHC1/11) to develop allosteric modulators that preserve enzymatic activity while blocking recognition of specific viral substrates; (3) employing PROTAC (proteolysis-targeting chimera) technology to selectively disrupt viral protein-enzyme complexes while sparing host interactions; and (4) developing host-directed therapies that specifically target virus-induced alterations in palmitoylation patterns rather than constitutive host enzymatic activity.

In conclusion, the in-depth investigation of the reversible S-palmitoylation and depalmitoylation system illuminates the core mechanisms of viral pathogenesis and host antiviral defense. Targeting palmitoylation dynamics offers an opportunity to modulate immune responses without broadly disrupting cellular signaling thereby offering a therapeutic potential. Collectively, these insights position reversible s-palmitoylation not merely as a molecular modification but rather as a regulatory system that helps understand predict and control host-virus interactions.
